# Television viewing and exposure to food-related commercials among European school children, associations with fruit and vegetable intake: a cross sectional study

**DOI:** 10.1186/1479-5868-4-46

**Published:** 2007-09-27

**Authors:** Knut-Inge Klepp, Marianne Wind, Ilse de Bourdeaudhuij, Carmen Perez Rodrigo, Pernille Due, Mona Bjelland, Johannes Brug

**Affiliations:** 1 Department of Nutrition, Faculty of Medicine, University of Oslo, Norway; 2 Department of Movement and Sport Sciences, Faculty of Medicine and Health Sciences, Ghent University, Belgium; 3 Unidad de Nutricion Comunitaria, Bilbao, Spain; 4 Department of Social Medicine, University of Copenhagen, Denmark; 5 EMGO Institute, VU University Medical Center, Amsterdam, the Netherlands

## Abstract

**Background:**

Fruit and vegetable intake is low among European children and exposure to TV is negatively associated with the intake of fruit and vegetables. The aim of the present study was to explore exposure to food commercials on TV in nine European countries. Associations between such exposure and intake of fruit and vegetables and possible mediating effects of attitudes toward and liking of fruit and vegetables were assessed.

**Methods:**

A cross-sectional survey was performed in nine European countries, i.e. Austria, Belgium, Denmark, Iceland, the Netherlands, Norway, Portugal, Spain and Sweden, from October-December 2003, as a part of the Pro Children study. Data on usual intake of fruit and vegetables, and related correlates were collected by means of a self-administered questionnaire among 11-year-old school children (mean age 11.4 (sd = 0.48), 50.2% boys). Complete data was available for 13,035 children. Differences in exposure to TV ads between countries, gender and social class were explored by analysis of variance. Multiple linear regression analysis was used to test associations between exposure to TV ads and intake and to assess mediating effects.

**Results:**

The large majority of children in all nine countries report recent exposure to a number of TV ads for food, and they were more often exposed to ads for unhealthy food than for fruit and vegetables (mean of 2.2 (sd = 1.0) unhealthy ads vs. mean of 1.7 (sd = 1.0) healthy ads; p < 0.001). Boys reported somewhat higher TV viewing than girls did (2.5 (sd = 1.7) vs. 2.2 (sd = 1.6) hours per day; p < 0.001), and children from lower social classes reported higher TV viewing than higher social class children did (2.4 (sd = 1.7) vs. 2.0 (sd = 1.5); p < 0.001). Across all countries, exposure to TV ads for healthy foods was positively associated (r = 0.09–0.16) with reported fruit and vegetable intake. This association was in part mediated by attitudes toward and liking of fruit and vegetables.

**Conclusion:**

Exposure to TV ads for fruit and vegetables appear to be associated with fruit and vegetable consumption among European school children. This relationship is in part mediated through cognitive factors such as attitudes and preferences concerning fruit and vegetables.

## Background

A majority of European school children across various geographical settings report eating less fruit and vegetables than recommended [1]. A comprehensive review of potential determinants of fruit and vegetable intake found a consistent, negative association between increased exposure to television (TV) viewing and reported consumption of fruit and vegetables across the four identified published papers investigating this association, i.e. for each additional hour of television viewed per day, fruit and vegetable servings per day decreased up to half a serving [2-6]. This finding was recently confirmed by data from the World Health Organization (WHO) cross-national study on health and health behaviors among adolescents in 35 countries (N = 162 305), where it was found that adolescent school children who watched more TV were less likely to eat fruit and vegetables daily [7]. Exposure to food advertising on TV is one of the proposed mechanisms for how TV viewing may impact children's eating behavior, and controlled studies have consistently demonstrated that children exposed to advertising choose advertised food products at significantly higher rates than those not exposed [8]. Food is the most frequently advertised product category on US children's television, and food ads account for over 50% of all ads targeting children [9]. A nutritional analysis conducted for the advertised foods in the UK found that 95% of the ads were for foods that were high in fat (62% of all ads), salt (61%) or sugar (50%) [10]. The results from international comparisons conducted as part of that analysis indicate that the advertising of high fat/high sugar foods to children is an international issue [10], and it has been suggested that comparative international studies could help shed light on the prevalence and impact of food marketing and advertising to children [9].

The purpose of the present study, which is part of a special series on Pro Children papers [11], was to investigate to what extent children across nine European countries report being exposed to food commercials on TV during the past month. Furthermore, we explored whether such exposure was related to intake of fruit and vegetables as reported by the children, and if so, to what extent this association was mediated by their attitudes toward and preferences for fruit and vegetable consumption.

## Methods

### Sample and procedure

The sample employed in this study is from the cross-sectional study of the Pro Children project involving nine European countries (Austria, Belgium, Denmark, Iceland, the Netherlands, Norway, Portugal, Spain and Sweden) [12]. The Pro Children study was designed to assess children's fruit and vegetable consumption, as well as potential determinants. A second objective was to develop, implement and test effectiveness of a school-based intervention in three of the Pro Children countries. For this study, data from the cross-sectional survey from all nine countries is used, which was conducted during October – December 2003 involving national representative samples of schools in all countries with the exception of Austria (for Austria, the sample was representative for the Eastern region) and Belgium (for Belgium, the sample was representative for Flanders). Schools were used as sampling unit, and from each country at least 20 schools were sampled and a minimum of 1,300 eligible children were included. Participation rates ranged from 70–97%, with Portugal (45%) and the Netherlands (30%) showing lower participation rates. Participating students completed a questionnaire in the classroom. Ethical approval was obtained from all relevant ethics committees in all countries, and parents of participating children as well as the children themselves gave their informed consent prior to participation.

Eleven-year old children were recruited to the study, and a response rate of 90.4% was reached in the participating schools; response rates ranged from 79.7–98.4%, with Portugal showing highest and the Netherlands showing lowest response rates. Mean age was 11.4 years (range 8.8–13.8, SD = 0.48; 79% of the children was born in 1992), and 50.2% were boys. The final sample sizes varied from 1,105 for the Netherlands to 2,134 for Portugal, with a total sample size of 13,305 students. A more detailed description of the Pro Children project, including the sampling and data collection procedure is given elsewhere [1,12].

### Questionnaire

A self-report questionnaire was developed to measure fruit and vegetable intake, and related correlates. The development of the questionnaire was based on theoretical models, a literature review, focus group interviews with children, individual interviews with parents and school staff, and thorough pre-testing [2,13-15]. In this report, the following variables are included:

#### Demographic data

sex (girl = 1 vs. boy = 2), age (calculated based on year and month of birth) and social class based on students' reports of their parents' occupation. This variable has been dichotomized into Social class I-II (1) vs. all others (0), including those with insufficient information to code occupation (Social class I: top managers, civil servants and educators usually having at least 4 years of university training; Social class II: other managers, medium level civil servants, primary school teachers and social workers). Students have been classified as belonging to Social class I or II if at least one of their parents' occupations was classified as such.

#### Exposure to TV viewing

Two items were included: "How often is the TV on during dinner (supper/evening meal) at home?" with response categories from 0 = never to 4 = every day (test-retest ICC = 0.76), and "About how many hours a day do you usually watch television and videos in your leisure time?" with response categories from 0 = none at all to 8 = about 7 hours a day or more. These two items were recoded to indicate number of days per week ('TV during dinner') and number of hours per day ('Regular TV viewing').

#### Exposure to TV commercials

Students were asked whether they, during the past month, had seen any TV commercials advertising 9 different kinds of foods: candy/chocolate bars; biscuits/sweet buns/cakes; fresh fruit; water; soda/soft drinks; vegetables; chips/savory snacks; fast food (e.g. burgers/hot dogs/French fries); or fruit juices. Response categories for each of these items were yes (1)/no (0). A composite 'TV ad exposure' score of all items was created (ranging from 0 to 9; (test-retest ICC = 0.67). Separate subscales were made for fruit, juice and vegetable ads ('FJV ads'; range: 0–3), and for sweet food items, i.e. chocolate & sweets, soft drinks and cakes & biscuits ('High sugar ads'; range: 0–3).

#### Attitudes toward fruit & vegetables

Two items assessing attitudes toward fruit and parallel items assessing attitudes toward vegetables were included: "To eat fruit [vegetables] every day makes me feel good" and "To eat fruit [vegetables] every day gives me more energy". These four items were all measured on a 5-point scale ranging from (1) fully agree to (5) fully disagree. Prior to the analyses, a composite score was calculated as the mean of these four items (Cronbach α = 0.81).

#### Liking fruit & vegetables (preferences)

As for attitudes, whether students liked fruit and vegetables or not was assessed with four items (same scale ranging from 1 to 5): "I like to eat fruit [vegetables] every day" and "Fruit [vegetables] tastes good" (Cronbach α = 0.73). Also for liking, a composite score was calculated as the mean of these four items.

#### Fruit and vegetable intake

Usual fruit and vegetable intake was measured using a food-frequency questionnaire. Children were asked how often they usually eat fresh fruit, drink 100% fruit juice, eat salad or grated vegetables, other raw vegetables and cooked vegetables. Response categories were (0) never, (1) less than one day per week, (2) one day per week, (3) 2–4 days a week, (4) 5–6 days a week, (5) every day, once a day, (6) every day, twice a day and (7) every day, more than twice a day. A separate validation study showed reasonable to good test-retest reliability (Spearman *r *from 0.47 to 0.84), and in general adequate validity comparing the food-frequency questions with 7-day food records (Spearman *r *from 0.40 to 0.53 for fruit and vegetable and 0.13 to 0.55 for fruit juice) [14]. Mean total frequency of fruit and vegetable intake per day was calculated by the sum of the frequency of intake of fresh fruit, 100% fruit juice, salad or grated vegetables, other raw vegetables and cooked vegetables, and used for the analyses presented in this paper.

### Statistical Analysis

Chi-square statistics was used to test the difference in proportions of male and females among drop-out and participants. Analysis of variance was used to test the differences in age and fruit and vegetable intake according to participation status, as well as differences in exposure to TV commercials between the countries, by gender and by social class (with adjustment for TV exposure and demographic factors). Pearson correlations coefficients were computed between all included variables. Multiple linear regression analysis was used to test the associations between exposure and intake, and whether or not such an association was mediated by the cognitive factors attitudes and liking. In the present study, mediation implies that, (I) TV exposure must be independently associated with the potential mediator (path a); (II) TV exposure must be associated with intake (path c); (III) the potential mediator must be independently associated with intake (path b), and (IV) the association between TV exposure and intake must decrease substantially when adjustment is made for the potential mediator. When testing all the paths we adjusted for sex, age, social class, TV viewing and TV viewing during dinner. Standardized regression-coefficients are presented. The above analyses were conducted for the total sample (across all countries), and for each country separately. Due to a large sample size and multiple tests, only p-values of 0.02 or less were seen as statistical significant and therefore 98% confidence intervals (CI) are reported. All analyses were conducted using SPSS 14.0.

## Results

Out of the 13,305 participating children, 532 did not respond to the item 'Regular TV viewing' which was located towards the end of the questionnaire, and they were omitted from the analyses presented in this paper. These 532 students were somewhat more likely to be boys than the remaining 12,773 participants (57.1% vs. 49.9%; χ^2 ^= 10.7; p = 0.001). These children were also slightly younger (11.3 vs. 11.4 years; F = 5.3; p = 0.02) and reported to eat fruit and vegetables less frequently (2.8 vs. 2.9; F = 6.2; p = 0.012) than the children that did respond to this TV exposure item.

Demographic data are presented by country in Table 1. Across countries, 24.4% of the children were found to come from families classified as belonging to social class I or II. Overall, participating children reported watching TV and videos 2.3 hours per day, and TV was on, on 3.4 days a week during dinnertime. Country differences were found regarding watching TV and videos (F = 37.2;p < 0.001). Post hoc test indicated that children from Belgium, the Netherlands and Portugal reported to watch TV and videos significantly more often than did children from the other countries (2.7 hours per day compared to 2.0–2.2 hours per day; Table 1). Boys reported significantly more TV viewing than girls (2.5 vs. 2.2 hours per day; F = 84.6; p < 0.001), and this pattern was seen across all countries, but did not reach statistical significance at the 2% level in the Netherlands, Norway and Sweden (data not shown). Children from families classified as belonging to social class I or II reported significantly less TV viewing than did children from lower social classes (2.0 vs. 2.4 hours per day; F = 130.6; p < 0.001), and this pattern was seen across all countries, but did not reach statistical significance at the 2% level for Iceland, Norway, Portugal and Sweden (data not shown).

**Table 1:** Demographic characteristics (sex, age, and social class) and TV exposure by country: The Pro Children study

	**Austria** (n = 1616)	**Belgium** (n = 1318)	**Denmark** (n = 1844)	**Iceland** (n = 1138)	**Netherlands** (n = 1094)	**Norway** (n = 1120)	**Portugal** (n = 2040)	**Spain** (n = 1272)	**Sweden** (n = 1331)
**Participation rate **(%)	95.3	84.5	92.0	88.7	79.7	89.5	98.4	94.7	84.2
**Sex: **% girls	53.0	46.1	49.1	47.8	53.8	49.6	53.1	46.4	50.0
**Age in years: **mean; 98%CI	11.0 10.97–11.03	11.5 11.43–11.49	11.4 11.36–11.41	11.3 11.24–11.30	11.7 11.67–11.75	11.3 11.27–11.34	11.5 11.47–11.51	11.4 11.39–11.45	11.4 11.35–11.41
**Social class: **% social class I-II	22.7	28.4	26.6	26.5	23.5	26.4	17.9	25.8	24.9
**Regular TV viewing (hours per day): ** mean; 98%CI	2.2 2.1–2.3	2.7 2.6–2.8	2.2 2.1–2.3	2.0 1.9–2.1	2.7 2.6–2.8	2.2 2.0–2.3	2.7 2.6–2.7	2.2 2.1–2.3	2.1 2.0–2.2
**TV during dinner (days per week)**: mean; 98%CI	2.8 2.7–3.0	2.9 2.7–3.0	2.3 2.2–2.5	4.7 4.5–4.9	2.5 2.3–2.7	1.9 1.7–2.1	5.5 5.4–5.7	4.8 4.6–5.0	2.8 2.6–2.9

We also observed large differences between countries with respect to having TV on during dinner (F = 358.8;p < 0.001). This was reported to be the case most days by children from Portugal and only seldom by children from Norway (5.5 vs. 1.9 days per week; Table 1). Overall, boys reported the TV to be on during dinner more days of the week than did girls (3.5 vs. 3.3; F = 14.6; p < 0.001), but this was significant only for the children from the Netherlands (F = 6.6; p = 0.01) and Spain (F = 9.6; p = 0.002). As for TV viewing, children from families classified as belonging to social class I or II reported the TV to be on during dinner significantly less than did children from lower social classes (2.7 vs. 3.7 days per week; F = 245.2; p < 0.001), and this pattern was consistent across all countries (data not shown).

As can be seen from Table 2, the proportion reporting having seen TV ads for various food items during the last month ranged from an overall high rate of 78% having seen ads for soda/soft drinks during the past month, to a low of 45% having seen ads for vegetables during the last month. Across all countries, a consistent pattern was seen in that the least frequently observed TV add was for vegetables, fresh fruit (the Netherlands) or fruit juices (Norway). The most frequently observed TV ads were for items high in sugar (soda/soft drinks and candy/chocolate) or high in fat (chips/savory snacks). TV ads for water were also frequently seen, particularly by children from Belgium, Portugal and Spain (Table 2).

**Table 2:** Proportion (%) reporting having seen TV ads during the previous month by country: The Pro Children study

	**Austria**	**Belgium**	**Denmark**	**Iceland**	**Netherlands**	**Norway**	**Portugal**	**Spain**	**Sweden**	**All**
Fresh fruits	72.8	54.9	58.1	63.8	48.6	68.1	56.7	59.9	51.6	59.5
Vegetables	52.5*	42.7	40.6	48.6	49.7	52.0	42.0	45.8	37.8	45.3
Fruit juices	76.6	66.3	64.5	80.2	70.9	46.2	60.0	83.5	44.1	65.6
Water	61.3	**81.3**	53.0	49.6	73.1	71.0	**75.9**	**90.2**	42.4	66.2
Candy/chocolate	77.4	77.9	**74.4**	60.5	81.4	77.2	65.4	84.3	66.7	73.5
Soda/soft drinks	78.4	80.8	72.1	73.9	**89.0**	80.0	75.8	88.1	73.4	**78.4**
Chips/savory snacks	79.7**	66.7	68.7	**82.9**	79.9	**82.2**	60.0	74.6	**84.0**	74.1
Fast food	66.7	56.5	63.6	78.8	73.2	72.7	69.4	86.1	78.7	71.0
Biscuits, sweet buns & cakes	55.2	53.5	50.7	62.9	64.1	59.1	61.8	83.3	67.5	61.3

* items with the lowest rate within each country are underlined

**items with the highest rate are marked in bold

In Table 3, the sum scores for TV ad exposure and the two sub-scales (FJV ads and High sugar ads) are presented by country. Overall, boys reported somewhat higher exposure to TV ads during the last month than did girls (6.1 vs. 5.8; F = 34.2: p < 0.001). Spanish children reported the highest level of exposure (7.0) and children from Denmark and Sweden the lowest level (5.5). In the total sample, children reported having seen more 'High sugar ads' than ads for fruit and vegetable (2.13; 98% CI: 2.11–2.15 vs. 1.70; 98% CI: 1.68–1.72) during the last month. In Table 3, reported exposure to the various TV ads, as well as the sum-scores assessing the children's attitudes and liking of fruit and vegetables are also presented by country. Overall, the children held positive attitudes and reported to like fruits and vegetables, the girls somewhat more than the boys (attitudes: 4.1 vs. 4.0; F = 45.4; p < 0.001 and liking: 4.1 vs. 3.9; F = 138.5; p < 0.001 for girls and boys respectively). Finally, in Table 3, the reported combined frequency intake of fruit and vegetable is presented by country. In the total sample, the girls reported to eat fruit and vegetable slightly more often than did the boys (3.1 vs. 2.9; F = 258.9; p < 0.001), and children from higher social class reported to eat fruit and vegetables slightly more often than did children from lower social classes (3.1 vs. 2.9; F = 101.6; p < 0.001).

**Table 3:** Exposure to TV ads, attitudes toward and liking of fruits and vegetables and usual intake: The Pro Children study

	**Austria**	**Belgium**	**Denmark**	**Iceland**	**Netherlands**	**Norway**	**Portugal**	**Spain**	**Sweden**
**TV ad exposure (number of TV ads seen during last month (0–9))**	6.2 6.1–6.3	5.8 5.7–5.9	5.5 5.3–5.6	6.0 5.9–6.2	6.3 6.2–6.4	6.1 5.9–6.2	5.7 5.6–5.8	7.0 6.8–7.1	5.5 5.3–5.6
**FJV ads (number of TV ads seen during last month (0–3))**	2.0 2.0–2.1	1.6 1.6–1.7	1.6 1.6–1.7	1.9 1.9–2.0	1.7 1.6–1.8	1.7 1.6–1.7	1.6 1.5–1.6	1.9 1.8–2.0	1.3 1.3–1.4
**High sugar ads (number of TV ads seen during last month (0–3))**	2.1 2.1–2.2	2.1 2.1–2.2	2.0 1.9–2.0	2.0 1.9–2.0	2.3 2.3–2.4	2.2 2.1–2.2	2.0 2.0–2.1	2.6 2.5–2.6	2.1 2.1–2.2
**Attitudes (mean of 4 items)**	4.2 4.2–4.2	3.8 3.7–3.8	3.9 3.9–4.0	4.0 3.9–4.1	3.8 3.7–3.8	4.1 4.0–4.1	4.4 4.4–4.5	4.1 4.1–4.2	4.0 3.9–4.0
**Liking (mean of 4 items)**	4.1 4.0–4.1	3.9 3.9–4.0	4.0 4.0–4.0	4.2 4.2–4.3	3.9 3.9–4.0	4.2 4.2–4.3	4.1 4.1–4.1	3.7 3.6–3.7	4.1 4.0–4.1
**Fruit and vegetable intake (mean of 5 items)**	2.9 2.9–3.0	3.2 3.2–3.3	3.0 2.9–3.0	2.9 2.8–3.0	3.0 2.9–3.1	2.9 2.9–3.0	3.1 3.1–3.2	2.9 2.9–3.0	2.9 2.8–3.0

mean values and 98% CI of the scales are presented

As can be seen from Table 4, all associations between the included variables were weak to moderate, with the exception of the potential mediators attitudes and liking which were strongly inter-correlated and also strongly associated with reported intake. Exposure to fruit and vegetable TV ads during the last month was weakly associated with attitudes toward fruit and vegetables and the children's liking (r = 0.15 for both scales), as well as to reported intake of fruit and vegetables (r = 0.12).

**Table 4:** Pearson's correlations between demographic variables, reported TV viewing, TV food ads exposure, attitudes and preferences concerning fruit and vegetable intake and reported intake. The Pro Children Study (n = 12,627)

	**1**	**2**	**3**	**4**	**5**	**6**	**7**	**8**
1 Age (mean in years)								
2 Sex (girl = 1, boy = 2)	.056							
3 Social class (low = 0, high = 1)	-.043	.010*						
4 Regular TV viewing (hours per day)	.107	.081	-.102					
5 TV during dinner (days per week)	.066	.034	-.138	.236				
6 FJV ads (number of TV ads seen last month; 0–3)	-.032	.010*	-.056	.014*	.059			
7 Attitudes toward fruit & vegetables (mean of 4 items; 1–5)	-.056	-.058	-.012*	-.105	.037	.154		
8 Liking fruit & vegetables (mean of 4 items;1–5)	-.032	-.102	-.021*	-.144	-.071	.149	.588	
9 Reported fruit and vegetable intake (mean of 5 items; 0–7)	.011*	-.139	.088	-.087	-.085	.124	.311	.439

* p > .001

**Figure 1:**
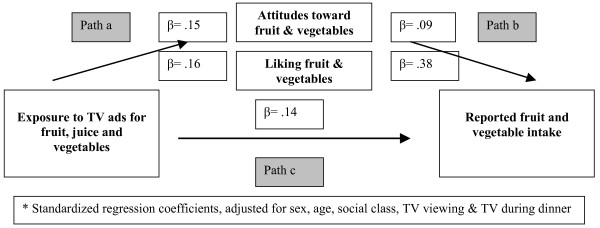
Conceptual model of the relationship between TV viewing, fruit and vegetable consumption and potential mediators: The Pro Children study

In Figure 1, the conceptual mediation model is depicted (i.e. paths a-c) along with the independent associations seen for each of these three paths. As the prerequisites for mediation clearly are met, as indicated by the standardized regression coefficients (for reported β, p < 0.001), we conducted the final analysis investigating the association between TV exposure and intake when adjusting for the potential mediators attitudes and liking. This model led to a substantial drop in the association between TV exposure and intake, i.e. from β = 0.14 to β = 0.07. Finally, the above analyses were conducted for each country separately. We found the adjusted association between TV ads and intake to be very stable across countries, as the observed standardized regression coefficients ranged from β = 0.06 to β = 0.08 for all countries with the exception of Norway (β = 0.12) and Spain (β = 0.04).

## Discussion

The results from this study demonstrates that the large majority of children in the nine European countries included in the Pro Children Study report watching TV, on average, more than 2 hours a day [16]. Boys and children from lower social class are more likely to watch TV than are girls and children from higher socio-economic backgrounds respectively. The differences seen in this study are consistent with those found in the WHO cross-national study on health and health behaviours among schoolchildren. While the WHO study included a broader age range than the Pro Children study, the findings concerning TV exposure by country, sex and socio-economic status are quite consistent across these two large studies [7,17].

Previous studies assessing the relation between watching TV and food intake [2-7] or the association between watching TV during dinner and the total amount of hours watching TV per day [18,19] have been reported. This study reveals quite large country differences regarding mealtime television viewing, as was published previously [16]. While watching TV during dinner seems to be more common in Portugal, Spain and Iceland, only few Norwegian children are used to watching TV during dinner. This might reflect differences among countries in how TV is perceived and used. Cultural differences, such as family food culture, parenting practices or the amount of advertisements on TV might mediate the influence of TV ads on children's intake. Further research should explore reasons for differences in TV watching habits during dinner. When addressing the main research questions of this study, i.e. the associations between different levels of exposure to TV ads and intake of fruit and vegetables and possible mediation by attitudes and preferences, analyses have been controlled for total TV viewing and watching TV during dinner.

This results from this study reveal that most children report seeing a number of TV ads for food, and that the children more frequently report seeing unhealthy food ads than ads for healthy foods such as fruit and vegetables. While watching TV, children are more exposed to unhealthy food items [9,10], and may therefore eat unhealthier. Several studies have shown that high exposure to TV ads is associated with increased food intake in children [20-23]. In the WHO health behavior in children study associations between intake and exposure to TV ads showed that the more children report to being exposed to TV ads, the less fruit and vegetables they consume daily [7]. In the current study we assessed associations between higher exposure to both healthy and unhealthy TV ads and intake of fruit and vegetables. A weak, but significant association was found between exposure to fruit and vegetable TV ads and reported intake of fruit and vegetables, i.e. the higher reported exposure to fruit and vegetable TV ads the more fruit and vegetables they reported to consume. Haerens et al. (2007) also revealed that children with better TV viewing behaviors consume more fruit [24]. Results from this study indicate that attention should be paid to how food is presented on TV and that it is important to portray healthy eating on children's TV programming. However, the present study is cross-sectional and we can therefore not establish causality. It may also be that children have greater attention for and recall of TV ads for foods they already eat more frequently.

While previous reports have concluded that TV commercials directed at children impact the children's purchasing and eating behavior [20-23], few studies have investigated what factors might mediate this observed behavior impact. The results from this study suggest that TV ads influence children's eating behavior by influencing their cognitions as expressed though attitudes toward fruit and vegetable consumption and preferences for these foods products. The direct relationship between TV ad exposure and reported intake was not eliminated but reduced in half in the final mediation analysis. Thus, a substantial amount of the association exercised by TV ads is mediated through attitudes and preferences, and this seems to hold through across a number of different cultural settings with large variations in TV viewing habits and eating habits [1,16,17,25]. Furthermore, the observed relationship was not a reflection only of exposure to TV overall, as this and another potential confounding factors was controlled for in the analysis.

The strength of the Pro Children study is first of all the employment of relatively large, representative samples of children and a carefully validated research instrument with a satisfactory measure of intake and measures of attitudes and preferences with good scale properties. Using a cross-sectional study design, however, limits our ability to establish causality between TV ads exposure and intake. Future analysis of data from the Pro Children cohorts established as part of the randomized, controlled intervention trials in the Netherlands, Norway and Spain [12], might contribute to further establish the temporal relationship between reported exposure and intake. The intake data used in this study has been validated and was regarded to be of acceptable validity [15]. Large sample sizes indeed might reveal statistically significant differences which are rather small. We therefore choose to use 98% confidence intervals. A more severe limitation of this study, however, is the rather crude measure used assessing fruit and vegetable TV ad exposure. Only whether or not the child had seen an ad over the past month was assessed. While the scale had good test-retest reliability, more detailed and validated measures of frequency and the content of the exposure, as well as exposure to food promotion through other channels would clearly have been preferable. The fact that a consistent relationship was observed even with this crude measure of exposure (with less than optimal variability across the sample) indicate the robustness both of the observed direct association and the mediated effects between TV exposure to fruit and vegetable promoting ads and reported intake.

## Conclusion

Children across European countries report exposure to food related TV ads; more so for unhealthy foods than healthy ones such as fruit and vegetables. Reporting being exposed to fruit and vegetable TV ads was found to be significantly and consistently positively associated with reported frequency of intake. This relationship was in part mediated through cognitive factors such as attitudes and preferences concerning fruit and vegetables. These findings point to the important role TV might exercise also when it comes to supporting healthy eating messages promoted at school and elsewhere. Prospective and intervention studies investigating the temporal and causal relationship between exposure and intake are, however, warranted prior to drawing firm conclusions concerning future intervention implications.

## Competing interests

The author(s) declare that hey have no competing interests.

## Authors' contributions

KIK preformed the statistical analysis, wrote the manuscript and incorporated inputs form all other authors on the manuscript. IDB, PD, CPR and JB participated in designing the study and project planning, and provided critical comments on the manuscript. MW and MB participated in the data collection and contributed in writing the manuscript. All authors have read and approved the final version of the manuscript.
